# Antioxidant Activity, Physicochemical and Sensory Properties of Stingless Bee Honey from Australia

**DOI:** 10.3390/foods13111657

**Published:** 2024-05-25

**Authors:** Mariana Mello dos Santos, Nazim Khan, Lee Yong Lim, Cornelia Locher

**Affiliations:** 1 Division of Pharmacy, School of Allied Health, The University of Western Australia, Crawley, WA 6009, Australia; mariana.mellodossantos@research.uwa.edu.au (M.M.d.S.); lee.lim@uwa.edu.au (L.Y.L.); 2 Department of Mathematics and Statistics, The University of Western Australia, Crawley, WA 6009, Australia; nazim.khan@uwa.edu.au

**Keywords:** stingless bee honey, Australia, physicochemical properties, antioxidant activity, sensory analysis, sugar content, trehalulose, high-performance thin-layer chromatography

## Abstract

This study reports on the physicochemical and sensory attributes, total phenolic content, and antioxidant activity of 36 honey samples produced by two different stingless bee species (*Tetragonula carbonaria* and *Tetragonula hockingsi*) from Australia. The findings reveal moisture content across all samples ranges from 24.9% to 30.8% (*w*/*w*), electrical conductivity from 1.02 to 2.15 mS/cm, pH levels between 3.57 and 6.54, soluble solids from 69.2 to 75.1 °Brix, trehalulose concentrations from 6.20 to 38.2 g/100 g, fructose levels from 7.79 to 33.4 g/100 g, and glucose content from 3.36 to 26.8 g/100 g. Sucrose was undetectable in all investigated samples. In a sensory analysis involving 30 participants, Australian stingless bee honey was perceived as having a more pronounced sourness compared with New Zealand Manuka honey. The study reveals considerable variability in the composition of Australian stingless bee honey, influenced by factors such as floral availability, geographical origin, and time of harvest. It also demonstrates the presence of phenolic compounds and antioxidant activity in stingless bee honey, underlining their potential as a natural source of antioxidants. All investigated samples contain trehalulose, which supports the findings of other recent studies that propose this unusual disaccharide as a marker compound of stingless bee honey.

## 1. Introduction

Honey is a complex natural product that is mainly produced from the nectar of flowers and processed by the digestive enzymes of a variety of bees. It has been used by humans since ancient times as a sweetener flavouring agent and also for its medicinal properties, particularly for wound healing [1]. There are two main types of commercially available honey: ‘common’ honey, produced by *Apis mellifera*, the European honey bee, and stingless or native bee honey, produced by various stingless bees. Stingless bees, as their name suggests, lack a functional sting and are characterised by their small, compact, dark-coloured appearance. There are approximately 500 species of stingless bees distributed throughout the tropical and subtropical regions of the world, such as Australia, Africa, Latin America, and Southeast Asia [2]. Eleven species of stingless bees (*Tetragonula* spp. and *Austroplebeia* spp.) are found within the tropical northern areas of Australia [3].

Due to their small size, stingless bees are excellent pollinators for crops that have small flower openings and are, therefore, difficult to access by the much larger European honey bees [4]. The honey they produce is a unique product that differs in colour, taste, and viscosity from honey produced by European bees. Its sweetness with an acidic undertone gives stingless bee honey a unique flavour profile [1].

Stingless bee honey contains more than 200 constituents, though it is composed mostly of sugars and water. It also contains small amounts of phenolic compounds, proteins (e.g., enzymes), amino acids, organic acids, mineral elements, and vitamins, which give the honey its distinct organoleptic characteristics [5,6]. It is important to highlight that the chemical composition of each type of honey is unique and directly related to several factors, including the particular stingless bee species involved in its production, its floral origin, environmental conditions, and the geographical location of the nectar source, as well as storage and processing conditions [7].

In Australia, stingless bee honey is also known as ‘sugarbag honey’ [8]. It has been a highly sought-after delicacy for First Nations Australians for thousands of years. The honey of stingless bees has also been traditionally used in indigenous cultures to treat skin conditions such as itchy skin and sores, and eating the bee brood was thought to be an effective treatment for common cold symptoms [9]. Although indigenous cultures have a long history of using stingless bee honey for a wide range of therapeutic purposes, research on the physico- and phytochemical properties of stingless bee honey and its various bioactivities remains limited, with a predominant focus to date on honey from Southeast Asia and Latin America [2,10,11,12,13]. To date, only a few studies have investigated the bioactivity of Australian stingless bee honey. Notably, these studies have found antimicrobial properties in honey produced by *Tetragonula carbonaria* [8,14,15]. However, the physicochemical properties and antioxidant activity of Australian stingless bee honey remain understudied.

A recent study discovered that trehalulose, a rare disaccharide, is present in abundance in stingless bee honey [16]. This unique sugar profile and other physicochemical characteristics of stingless bee honey do not conform to current international and national food standards for honey. More research is, therefore, required to establish appropriate standards specifically for stingless bee honey [17] to ensure the quality and authenticity of stingless bee honey.

Given the sparsity of data on Australian stingless bee honey, the aim of this study is to investigate the antioxidant activity and the physico- and phytochemical, as well as olfactory and gustatory, properties of various honey samples produced by the two most domesticated Australian stingless bee species, *Tetragonula carbonaria* and *Tetragonula hockingsi*.

## 2. Materials and Methods

### 2.1. Reagents and Solutions

The chemicals and reagents used in this study and their suppliers are as follows: trehalulose (Biosynth Carbosynth, Staad, Switzerland); fructose, sucrose, and sodium carbonate anhydrous (Chem-Supply Pty Ltd., St. Gillman, SA, Australia); anhydrous sodium acetate, glucose, gallic acid, and phosphoric acid (Ajax Finechem Pvt Ltd.s., Cheltenham, VIC, Australia); boric acid (Pharma Scope, Welshpool, WA, Australia); maltose, trolox, Folin–Ciocalteu Phenol reagent 2N, 2,4,6-tris(2-pyridyl)-1,3,5-triazine (TPTZ), iron (III) chloride hexahydrate, iron (II) sulphate heptahydrate (Sigma-Aldrich, St. Louis, MO, USA); diphenylamine (The British Drug Houses Ltd., London, UK); aniline (Fluka AG, Buchs, Switzerland); and 2,2-diphenyl-1-picrylhydrazyl (DPPH) (Thermo Fisher Scientific, Ward Hill, MA, USA).

Solvents and their suppliers are as follows: methanol and 2-propanol (Merck KGaA, Darmstadt, Germany), 1-butanol (ChemSupply Pty Ltd., St. Gillman, SA, Australia), and glacial acetic acid (Ajax Finechem Pvt Ltd.s., Cheltenham, VIC, Australia).

Silica gel 60 F254 HPTLC glass plates (20 cm × 10 cm) were obtained from Merck KGaA (Darmstadt, Germany).

### 2.2. Honey Samples

For this study, a total of thirty-six stingless bee honey samples, of which twenty-eight were produced by *Tetragonula carbonaria* and eight samples from *Tetragonula hockingsi*, were obtained directly from local beekeepers in Queensland, Australia (Table 1). They were all harvested between 2020 and 2022. Samples considered natural duplicates in this study (*n* > 1) were harvested at the same time from different hives located on the same apiary site. New Zealand Manuka honey (100% pure New Zealand Honey^®^), used as a comparator honey in the study’s organoleptic assessment, was purchased from an online shop. All samples were stored at 4 °C until further analysis.

### 2.3. Physicochemical Analysis

#### 2.3.1. Soluble Solids (°Brix) and Moisture

A digital refractometer (Hanna Instruments, HI96801, Woonsocket, RI, USA) was used to determine the total soluble solids in the honey samples. A small amount of each honey sample was applied to the refractometer’s glass prism, and the reading was recorded at room temperature. Results were expressed in °Brix [18].

By adopting the same analytical approach, honey moisture was expressed as (100%-°Brix).

#### 2.3.2. Electrical Conductivity

The electrical conductivity of 20% (*w*/*v*) aqueous honey solutions was determined using a waterproof pH/EC/TDS/temperature tester (Hanna instruments, HI98131, Woonsocket, RI, USA). Results were expressed in mS/cm [19].

#### 2.3.3. pH

The pH of solutions of 1 g honey dissolved in 7.5 mL of deionised water was measured at room temperature using a pH meter (Oakton, pH 700, Singapore) [18].

### 2.4. Sugar Analysis

High-Performance Thin-Layer Chromatography (HPTLC) was used for the identification and quantification of the main sugars present in the honey samples. Standard solutions were prepared in 50% aqueous methanol for each sugar at the following concentrations: trehalulose (200 µg/mL), glucose (250 µg/mL), fructose (250 µg/mL), and sucrose (100 µg/mL). Honey solutions were prepared by dissolving 100 mg of honey in 100 mL of 50% aqueous methanol.

The mobile phase used was composed of 1-butanol/2-propanol/aqueous boric acid (5 mg/mL) at a ratio of 30:50:10 (*v*/*v*) [20,21]. To prepare the derivatisation reagent, 2 g of diphenylamine and 2 mL of aniline were dissolved in 80 mL of methanol, followed by the addition of 10 mL of phosphoric acid (85%). The solution was made up to 100 mL using methanol.

The chromatographic analysis was conducted using a semi-automated HPTLC application device (Linomat 5; CAMAG, Muttenz, Switzerland). Calibration curves for each standard solution were generated at the following concentration ranges: trehalulose (100–800 ng/band), fructose (250–1250 ng/band), glucose (250–1250 ng/band), and sucrose (100–500 ng/band). The application volume for each honey solution was adjusted to provide sugar concentrations that fit the standard curves; volumes ranged from 0.5 to 7 µL. Standard sugar and honey solutions were applied on an HPTLC plate at a rate of 40 nL/s at 8.0 mm from the base and 20.2 mm from the side edges of the plate. Band lengths of 8.0 mm were applied with an 11.4 mm distance between the bands. HPTLC plates were developed at ambient temperature with the mobile phase in a saturated (33% relative humidity) automated development chamber (ADC2, CAMAG). The development chamber was saturated for 60 min, the plates were pre-conditioned with the mobile phase for 5 min, and 10 mL of mobile phase were used for development to a migration distance of 85 mm. After development, the plates were dried for 5 min. An HPTLC imaging device (TLC Visualiser 2, CAMAG) was used to document the chromatographic results under white light. The HPTLC software (visionCATS v3.1, CAMAG) was used to analyse the documented images. After development, the plates were derivatised with 2 mL of an aniline-diphenylamine-phosphoric acid reagent using a TLC derivatiser (CAMAG Derivatiser, yellow nozzle). Afterwards, the plates were heated for 10 min at 115 °C using CAMAG TLC Plate Heater 3. After cooling the plates to room temperature, the HPTLC imaging device was again used to analyse the plates under transmission white (T white) light [20,21]. Honey solutions were prepared in triplicates, and results were expressed as the average amount of sugar (in g) per 100 g of honey ± standard deviation.

### 2.5. Consumer Evaluation

The consumer evaluation protocol was approved by the University of Western Australia Human Research Ethics Committee (2022/ET000786), and informed written consent was obtained from all participants. Volunteer participants consisted primarily of students and staff of the University of Western Australia. A total of 30 participants (11 males and 19 females) were recruited, with an average age of 28.6 ± 8.51 years (ranging from 19 to 58 years).

Prior to the sensory evaluation, participants fasted for two hours to ensure that taste perception remained unaffected [22]. Additionally, participants were instructed to avoid using perfumed toiletries to minimise potential interference in the evaluation of the olfactory properties of the selected honeys. Five honey samples in total were assessed in each evaluation session: four samples of Australian stingless bee honey (two samples from group C1, referred to as C1a and C1b, along with one sample from group C2 and one sample from group H1) and one sample of New Zealand Manuka honey. The stingless bee honey samples were selected to ensure the representation of different stingless bee species.

Each participant completed a preliminary test to assess their ability to determine different levels of sweetness and sourness. During the preliminary test, participants were presented with solutions containing white sugar at different concentrations (1, 100, and 350 g/L) and asked to rank them from the most to least sweet. Five minutes later, a similar test was conducted where participants received lemon juice diluted with water at 25-, 50-, and 100-fold dilution and were asked to rank these solutions from most to least sour [23].

**Figure 1:**
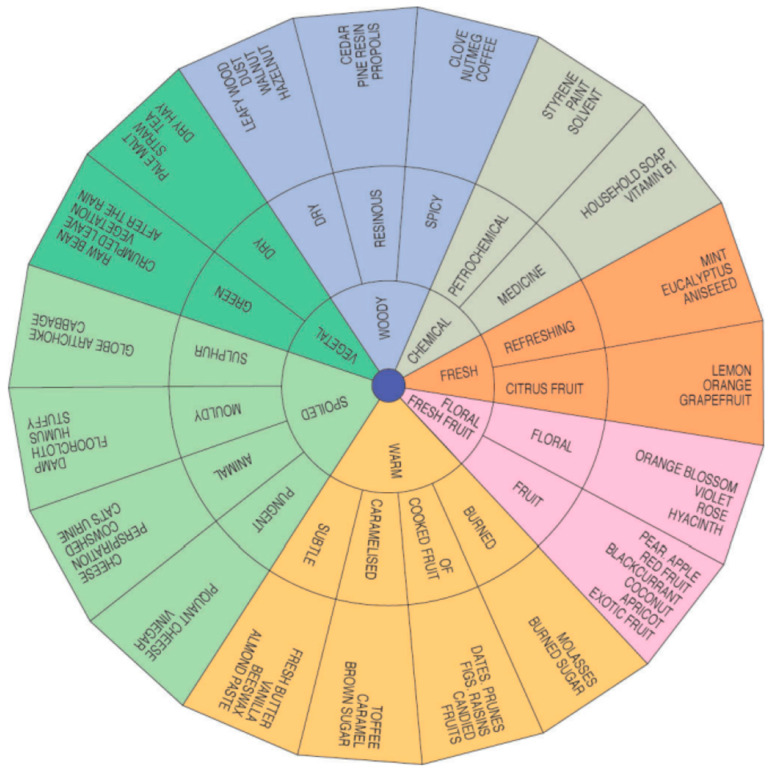
Odour and aroma wheel (reprinted from ref. [22]).

After the preliminary test, participants received closed glass jars containing 3 mL of each of the five de-identified honey samples, which were presented in randomised order. Following the opening of the sample jars, the participants immediately assessed the olfactory characteristics, including odour intensity, persistence, and odour attributes. These attributes were evaluated based on an odour and aroma wheel (Figure 1), as described by Piana et al. [22]. Participants used a rating scale ranging from 1 to 3 to indicate the intensity and persistence of the aroma, with 1 representing low intensity/persistence and 3 representing high intensity/persistence.

The taste characteristics were evaluated by providing participants with honey samples on disposable spoons. Between each honey sample, participants consumed sips of water to cleanse their palates, ensuring that no residual honey taste affected subsequent assessments. Participants reported the perceived sweetness and sourness of each sample on a scale from 1 to 5, where 1 indicated not sweet/sour, and 5 indicated extremely sweet/sour.

Finally, participants were asked to express how much they liked each honey sample based on the respective olfactory and taste characteristics on a scale from 1 to 5, with 1 being the least liked and 5 being the most liked sample [24].

The R statistical environment was used for all data analyses. A linear mixed model was fitted to the scores for each trial (aroma, sweetness, and sourness) to estimate any differences in mean scores for the five honey types after adjusting for demographic variables. Given that participants may have varying perceptions of sweetness and sourness, a random intercept term for participants was included to accommodate these differences. The model allows for the identification of any discrepancies between participants and accounted for them in the analysis.

### 2.6. Determination of Total Phenolic Content (TPC)

The total phenolic content in the honey samples was determined using the colourimetric Folin–Ciocalteu assay, following the methodology described by Liberato et al. [25] with minor modifications. An artificial honey solution was prepared by mixing 21.63 g of fructose, 18.13 g of glucose, 1.00 g of maltose, 0.75 g of sucrose, and 8.50 g of water [26]. Solutions of gallic acid ranging from 0.06 mg/mL to 0.18 mg/mL were used to derive a standard curve.

Samples were prepared as follows: (a) 200 µL of each aqueous honey solution (20% *w*/*v*), (b) 100 µL of each gallic acid standard spiked with 100 µL artificial honey (40% *w*/*v*), and (c) 100 µL water spiked with 100 µL artificial honey (40% *w*/*v*) (blank). Each sample was reacted with 1 mL of a diluted Folin–Ciocalteu reagent (1 mL of Folin–Ciocalteu reagent in 30 mL of deionised water). After 5 min, 800 µL of 0.75% Na_2_CO_3_ solution was added. The mixture was left to incubate in the dark for 2 h. The absorbance was recorded at 760 nm using a spectrophotometer (Agilent Technologies, Cary 60 UV-Vis Spectrophotometer, Santa Clara, CA, USA). The analysis was carried out in triplicate, and the mean result for each sample was expressed as mg gallic acid equivalent (GAE) per 100 g of honey ± standard deviation. The total phenolic content was calculated with Equation (1) as follows:

(1) TPC(mgGAE)=(ΔAbs−intercept)/slope

### 2.7. Determination of Antioxidant Activity Using the Ferric Reducing Antioxidant Power (FRAP) Assay

The antioxidant capacity of the honey samples was assessed using the Ferric Reducing Antioxidant Power (FRAP) assay. This assay relies on the reduction of ferric 2,4,6-tris(2-pyridyl)-1,3,5-triazine [Fe(III)-TPTZ] to a ferrous complex under acidic conditions, followed by spectrophotometric analysis. The assay was conducted according to the protocol described by Almeida et al. [27] with slight modifications.

Briefly, the FRAP reagent was prepared by mixing 10 mM TPTZ (dissolved in 40 mM HCl), 20 mM aqueous FeCl_3_·6H2O, and 300 mM aqueous acetate buffer (pH 3.6) in a 1:1:10 (*v*/*v*/*v*) ratio. This reagent mixture was freshly prepared for every experiment and allowed to incubate at 37 °C before use. Ferrous sulphate (FeSO_4_·7H_2_O) standard solutions (200 µM to 1200 µM) were freshly prepared to produce a standard curve. A standard solution of 600 µM ferrous sulphate was used as a positive control.

Samples were prepared by mixing 20 µL of aqueous honey solution (20% *w*/*v*) with 180 µL FRAP reagent in a 96-well microplate (Greiner Bio-One 96-well Microplate Flat Bottom). The reaction mixtures were incubated in the dark for 30 min, and their absorbance was measured at 620 nm using a POLARstar Optima Microplate Reader (BMG Labtech, Allmendgrün, Ortenberg, Germany). The analysis was carried out in triplicate, and the mean FRAP activity for each sample was expressed as mmol Fe^+2^/kg fresh weight of honey ± standard deviation. The FRAP antioxidant activity was calculated using Equation (2) as follows:

(2) FRAP(µMFe(II))=(ΔAbs−intercept)/slope

### 2.8. Determination of Antioxidant Activity Using the 2,2-Diphenyl-1-Picrylhydrazyl (DPPH) Radical Scavenging Assay

The antioxidant activity of the honey samples was also assessed with the DPPH assay, which measures the antioxidant scavenging ability of 2,2-diphenyl-1-picrylhydrazyl. To prepare the DPPH reagent mixture, a methanolic DPPH solution at 0.130 mM was mixed with aqueous acetate buffer (100 mM, pH 5.5) at a ratio of 19:10 (*v*/*v*). Aqueous Trolox solutions (100 to 600 µM) were prepared to produce a standard curve. The Trolox solutions had their pH adjusted to 7.0. A standard solution of 400 µM Trolox was used as a positive control [28].

Samples were prepared by mixing 10 µL of an aqueous honey solution (20% *w*/*v*) with 290 µL of DPPH reagent in a 96-well microplate (Greiner Bio-One 96-well Microplate Flat Bottom). The reaction mixture was kept in the dark for 2 h before the absorbance was measured at 520 nm using a POLARstar Optima Microplate Reader (BMG Labtech, Allmendgrün, Ortenberg, Germany). The analysis was carried out in triplicate, and the mean DPPH activity for each sample was expressed as mmol Trolox Equivalent (TE)/kg fresh weight of honey ± standard deviation. The DPPH antioxidant activity was calculated using Equation (3) as follows:

(3) DPPH(µMTrolox)=(ΔAbs−intercept)/slope

### 2.9. Statistical Analysis

Significant differences between samples were determined using analysis of variance (ANOVA) followed by post-hoc Tukey for the comparison of means. All statistical analyses were performed using the IBM SPSS Statistical package. A *p*-value < 0.05 was considered to be significant.

## 3. Results and Discussion

### 3.1. Physicochemical Parameters

The physicochemical characteristics (i.e., moisture, soluble solids, electrical conductivity, pH) of the stingless bee honey samples are presented in Table 2.

With the exception of Heather honey, the *Codex Alimentarius* requires honey to have a moisture content of no more than 20% [29]. However, stingless bee honey typically contains higher moisture levels when compared to *Apis mellifera* honey [30]. This difference can be attributed to the humid tropical environment in which these bees operate, resulting in the collection of nectar, which naturally contains higher levels of moisture [31]. Other contributing factors include nectar sourced from undergrowth flowers and the involvement of various bee species [31]. In this study, the average moisture content of the honey samples was determined to be 27.6% ± 1.26, with the lowest value observed in sample H4 (24.9%) and the highest value in sample H1 (29.9%), both honeys produced by *Tetragonula hockingsi*. These values are in agreement with published data. Oddo et al. [32], who investigated Australian *Tetragonula carbonaria* honey, reported moisture values ranging from 25.3% to 27.5%. Zawawi et al. [17] analysed the moisture content in Australian stingless honey produced by *Tetragonula carbonaria* and *Tetragonula hockingsi* and found moisture values between 23.8% and 27.7%. Honey from stingless bees in Brazil [33] and Thailand [2] exhibited average moisture values of 30.7% and 31.0%, respectively.

Total soluble solids in honey are correlated with its moisture and sugar content [34]. Typically, honey with high levels of total soluble solids contains elevated sugar levels and a lower moisture content. The average soluble solid content measured in the honey samples was 72.4 ± 1.26 °Brix, with values ranging from 70.2 to 75.1 °Brix. Among these samples, H1 (produced by *Tetragonula hockingsi*) displayed the lowest value, while H4 (also produced by *Tetragonula hockingsi*) had the highest. Biluca et al. [33] reported similar results for stingless bee honey samples from Brazil, with levels ranging from 60.7 to 74.6 °Brix. In honey produced by *Apis mellifera*, the °Brix value is commonly higher compared with what is typically observed in stingless bee honey, primarily due to stingless bee honey’s higher water level and correspondingly lower sugar content [34].

The electrical conductivity of honey is directly linked to its concentrations of minerals, salts, organic acids, and proteins [35]. Across the honey samples examined in this study, the average electrical conductivity was found to be 1.52 ± 0.227 mS/cm, while the range was from 1.24 (sample C6, produced by *Tetragonula carbonaria*) to 2.15 (sample C8, produced by *Tetragonula carbonaria*) mS/cm. Notably, only one sample (C8) had a conductivity exceeding 2.00 mS/cm. This finding aligns with the observations by Oddo et al. [32], who reported an average electrical conductivity of 1.64 ± 0.12 mS/cm in Australian stingless bee honey. Similarly, Chuttong et al. [2] documented comparable results for stingless bee honey from Thailand, with an average of 1.1 ± 0.780 mS/cm. In contrast, Zawawi et al. [17] found lower electrical conductivity values for *Tetragonula carbonaria* and *Tetragonula hockingsi* honey from Australia, reporting average values of 0.61 ± 0.11 and 0.70 ± 0.05 mS/cm, respectively. According to Nordin et al. [36], who conducted a comprehensive review of the physicochemical properties of stingless bee honey globally, the electrical conductivity of such honey varies widely, ranging from 0.102 mS/cm to 8.77 mS/cm, with an average of 1.16 ± 0.16 mS/cm. This broad range indicates substantial variability among stingless bee honey samples. The findings of this study highlight that the International Codex Alimentarius Commission guidelines for honey, which require an electrical conductivity of less than 0.8 mS/cm for most kinds of honey, except for those produced from honeydew, chestnut flowers, and mixtures of these [29], might not be suitable for all stingless bee honeys.

The pH of honey can be influenced by various factors, including the source of the flower nectar collected for honey production, the timing of the harvest, and concentrations of different acids [37]. The majority of the honey samples in this study exhibited acidic characteristics, although a large range in pH values, from 3.60 to 5.90, was observed (Table 2). Sample C4 (produced by *Tetragonula carbonaria*) registered the lowest pH value, while sample H2 (a *Tetragonula hockingsi* honey) recorded the highest. The average pH value for all analysed samples was 4.26 ± 0.757, similar to the average pH of 4.00 reported by Oddo et al. [32] for Australian stingless bee honey. Similar pH values have also been documented for Brazilian stingless bee honey, ranging from 3.16 to 6.56 [33]. In contrast, honey from stingless bees in Thailand was found to be more acidic, with an average pH value of 3.60 [2]. Interestingly, Zawawi et al. [17] also reported a more acidic pH range, from 3.44 to 3.88, for honey produced by *Tetragonula carbonaria* and *Tetragonula hockingsi* species in Australia.

### 3.2. Sugar Analysis

Stingless bee honey exhibits a distinctive sugar profile primarily due to the abundant presence of trehalulose, a rare disaccharide recently identified in stingless bee honey from various regions that has been, therefore, proposed as a marker compound for this kind of honey [16]. Trehalulose is considered important for distinguishing between stingless bee honey and honey produced by European honey bees. The latter typically consists of fructose as the most abundant sugar (approximately 32–38%), followed by glucose. In contrast, stingless bee honey is primarily characterised by the presence of the disaccharide trehalulose [16,38].

**Figure 2:**
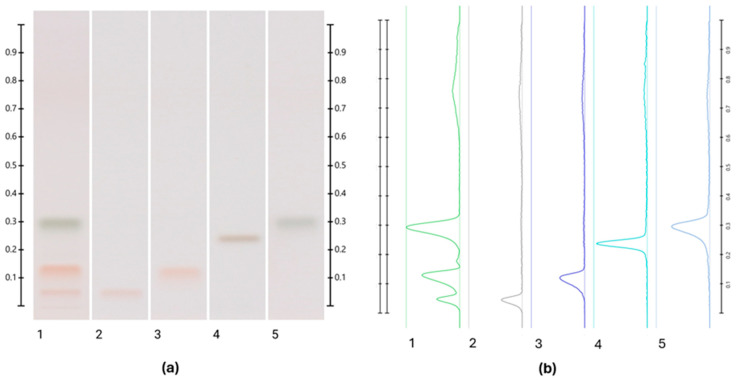
An example of an HPTLC chromatogram showing the separation of sugars. Images were taken at T white light; (**a**) Track 1—honey sample, track 2—trehalulose standard, track 3—fructose standard, track 4—sucrose standard, and track 5—glucose standard; (**b**) their respective chromatograms.

The qualitative and quantitative assessment of Australian stingless bee honey samples’ main sugars, including trehalulose, fructose, glucose, and sucrose, was conducted using High-Performance Thin-Layer Chromatography (HPTLC) [20,21], which is a convenient method for distinguishing between the sugars based on their retardation factor (Rf) and band colours (Figure 2).

Table 3 summarises the sugar profiles and fructose-to-glucose (F/G), fructose-to-trehalulose (F/T), and glucose-to-trehalulose (G/T) ratios of the analysed honey samples. All honey samples predominantly consisted of trehalulose and fructose, with lesser amounts of glucose. Trehalulose, in line with the literature, was present in all samples, averaging 18.1 ± 7.17 g/100 g honey. Sample H3 (from *Tetragonula hockingsi*) exhibited the lowest trehalulose content (10.0 g/100 g honey), while sample C2 (from *Tetragonula carbonaria*) had the highest (30.7 g/100 g). Although trehalulose was present in all samples, which is consistent with findings in the literature, it was not the predominant sugar in every sample. The results indicate significant differences (*p* < 0.05) among honey samples harvested from the same district area. Specifically, honey samples collected in May generally exhibited higher levels of trehalulose compared with those harvested in September and November. Additionally, honey samples collected in September had higher trehalulose levels than those collected in November. Excluding samples C7, C8, H4, and H5, which are from a different, district area, the average trehalulose content for samples collected in May was 28.1 ± 5.18 g/100 g, while honey samples harvested in September and November averaged 17.0 ± 4.00 and 12.0 ± 3.06 g/100 g of trehalulose, respectively. This suggests that factors such as harvest time and flower seasonality may significantly influence the trehalulose content in stingless bee honey samples. In contrast, Zawawi et al. [17] found trehalulose to be the primary sugar in all examined Australian and Malaysian stingless bee honey samples, with concentrations ranging from 17.8 to 57.0 g/100 g. A study conducted by Oddo et al. [32] examined honey produced by *Tetragonula carbonaria* exclusively and determined the sugar composition using High-Performance Liquid Chromatography (HPLC). This study identified an unconventional sugar composition when compared to *Apis mellifera* honey, with the stingless bee honey featuring approximately 20% maltose. However, the retention time of what the study tentatively identified as maltose did not perfectly match that of the maltose standard. This discrepancy strongly suggests that the sugar in question is more likely to be trehalulose. With this, the findings of Oddo et al. are also in broad agreement with the trehalulose levels determined in this study.

**Figure 3:**
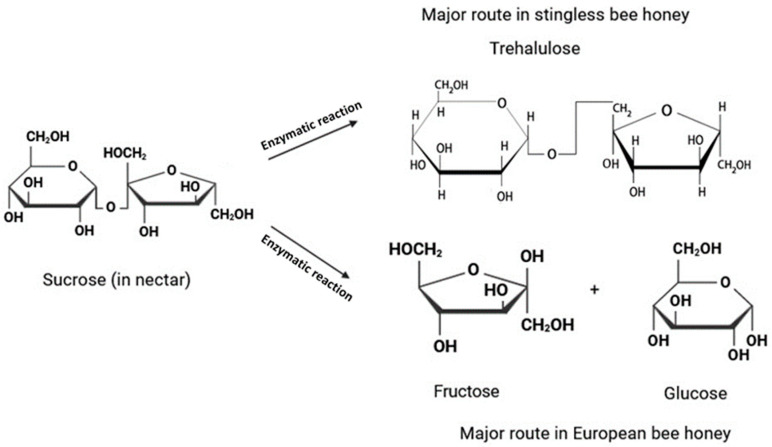
Formation mechanism of trehalulose, fructose, and glucose in honey (adapted with permission from ref. [39]. Copyright 2022 American Chemical Society).

The average fructose content was 22.6 g/100 g ± 7.11, with samples C2 (10.2 g/100 g), produced by *Tetragonula carbonaria*, and H3 (31.1 g/100), produced by *Tetragonula hockingsi*, showing the lowest and highest amounts, respectively. Glucose content ranged from 4.31 g/100 g (sample C8, produced by *Tetragonula carbonaria*) to 24.0 g/100 g (sample H3, produced by *Tetragonula hockingsi*), with an average value of 15.3 ± 6.72 g/100 g. Notably, a strong positive correlation factor of 0.962 was observed between fructose and glucose. However, both fructose and glucose exhibited negative correlations with trehalulose (−0.966 and −0.937, respectively), indicating that honey samples with high trehalulose content displayed low levels of fructose and glucose and vice versa. These correlations are in line with the proposed biochemical pathway of trehalulose formation in stingless bee honey, as shown in Figure 3.

Sucrose was not detected in any of the analysed samples. The absence of sucrose supports the hypothesis that trehalulose formation involves an enzymatic reaction where sucrose occupies the enzyme’s active site, acting as a donor to form a β-D-glucosyl-enzyme intermediate. Upon releasing a fructose moiety, this glucosyl−enzyme intermediate then binds to either the same or another free fructose moiety at 1-OH to form trehalulose [39] (Figure 3). Hence, variations in trehalulose content could be attributed to differences in the amount of sucrose present in the nectar collected by the bees, as lower sucrose levels in the nectar result in reduced availability for conversion into trehalulose [38].

Furthermore, the crystallisation of honey is closely associated with the ratio of fructose to glucose (F/G), more specifically, the different solubilities of the two sugars. When the F/G ratio falls below 1.00, honey tends to crystallise rapidly, while a ratio exceeding 1.00 keeps honey in a liquid state for an extended period [40]. In addition, the rate of crystallisation in honey is also influenced by the glucose/water ratio. Therefore, moisture levels in honey play an important role in crystallisation [40]. In all stingless bee honey samples analysed as part of this study, an F/G ratio greater than 1.00 was observed, ranging from 1.22 (sample C5 from *Tetragonula carbonaria*) to 2.56 (sample C8 from *Tetragonula carbonaria*). Coupled with their relatively high moisture content, this explains the samples’ low tendency to crystallise. The ratios of fructose to trehalulose (F/T) and glucose to trehalulose (G/T) were also determined, ranging from 0.331 to 3.09 and 0.150 to 2.39, respectively. Sample C2, produced by *Tetragonula carbonaria*, exhibited the lowest ratios for both F/T and G/T, while sample H3 from *Tetragonula hockingsi* displayed the highest ratios for both comparisons.

The analysis of the sugar profile of honey holds significant importance in detecting potential adulteration and assessing honey quality. The absence of detectable levels of sucrose in the analysed samples indicates a lack of adulteration, as high sucrose content may signal the addition of commercial sugar [41]. Nevertheless, it is important to highlight that the detection of adulteration in honey should not rely solely on its sugar profile. This is because when sucrose is fed to stingless bees, they might convert sucrose into trehalulose, resulting in honey with a similar sugar profile to that found naturally; however, it likely lacks other essential components that contribute to the overall quality of honey [42].

Moreover, in high-quality honey, the glucose content typically falls below that of fructose, a characteristic observed in all analysed stingless bee honey samples [43]. The presence of trehalulose, no matter whether at high or low levels, is also an important point of consideration. Given its established status as a marker compound for stingless bee honey independent of their geographical location, it plays an important role in distinguishing stingless bee honey from honey produced by *Apis mellifera* bees. While in rare instances, the latter might contain very low concentrations of trehalulose, its presence is mandatory for any honey claimed to be produced by stingless bees. In this way, trehalulose might assist in the authentication of stingless bee honey. This view has been supported by findings of this study where trehalulose, albeit in varying concentrations, has been detected in all stingless bee honey, even when produced by different stingless bee species (i.e., *T. carbonaria* and *T. hockingsi*).

### 3.3. Consumer Evaluation

A sensory evaluation is a simple, low-cost method used to describe the organoleptic characteristics of a product and evaluate its acceptability by consumers. While sensory analysis has been extensively employed to describe the taste and odour attributes of European bee honey [23,24] and even stingless bee honey from various other regions [44], it appears that, to date, there is a lack of studies evaluating the taste and aroma profiles of Australian stingless bee honey, especially in comparison with ‘common honey’.

**Figure 4:**
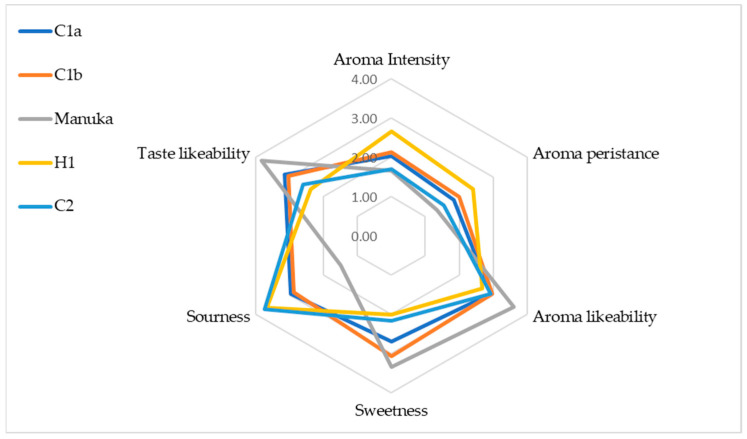
Mean values for the honey sensory analysis (honey sample *n* = 5).

As can be observed in Figure 4, the stingless bee honey used in this study had a more intense and persistent aroma compared with the New Zealand Manuka honey. In terms of sourness, stingless bee honey samples were also found to be sourer than the comparator honey. Samples C2 and H1 were the sourest, with mean scores of 3.73 and 3.67, respectively. In contrast, Manuka honey showed the lowest sourness, with a mean score of 1.50. This observation is in line with the findings of other studies, which also attributed the distinctive sour taste of stingless bee honey to its high content of polyphenolic compounds, particularly phenolic acids. The phenolic acids are likely also responsible for the low pH observed in most stingless bee honey [1,45].

When evaluating sweetness, stingless bee honey samples demonstrated reduced levels of sweetness in comparison to Manuka honey. Manuka honey showed the highest mean sweetness score (3.33), while samples H1 (2.00) and C2 (2.17) registered the lowest mean sweetness scores.

The findings of this sensory evaluation align with previous research that also found a distinguishable difference in the taste of honey produced by stingless bees and European honey bees. Next to their differences in pH, this might also be related to the honey’s sugar profile. As mentioned previously, stingless bee honey commonly contains significant amounts of trehalulose and lower levels of glucose and fructose, whereas ‘common’ honey’s primary sugars are glucose and fructose.

Participants were also tasked with assessing the odour attribute of the five samples by referencing the odour and aroma wheel of honey. About one-fifth of participants noted a pungent odour in samples C1a and H1 (16.7 and 20%, respectively), while 16.7% claimed that sample C1b had a pungent odour along with an aroma of cooked fruit, whereas another 16.7% of participants thought that sample C2 had an odour reminiscent of cooked fruit. Regarding Manuka honey, about one-quarter of participants (23.3%) perceived it to have a refreshing odour.

Overall, participants showed a preference for the taste and aroma of Manuka honey, giving it mean scores of 3.83 and 3.60, respectively. In contrast, sample H1 had the lowest acceptance, with a taste mean score of 2.37 and an aroma mean score of 2.67.

### 3.4. Total Phenolic Content (TPC)

The Folin-Ciocalteu (FC) assay is a widely employed method for determining the total phenolic content (TPC) in food and plant products, beverages, honey, and other natural products. However, the presence of reducing sugars in honey can introduce interference, potentially leading to an overestimation of TPC. In this study, a modified Folin–Ciocalteu method was employed to minimise this impact [26]. In the modified assay protocol, a pH of 7.9 was maintained, achieved by using a 0.75% aqueous Na_2_CO_3_ solution, which enables the accurate measurement of phenolic compounds without interference from reducing sugars. In addition, the spectrophotometer was blanked using an artificial honey solution. Given the scarcity of studies on the sugar profile of stingless bee honey, especially from Australia, it was not feasible to prepare a ‘typical’ stingless bee artificial honey. Consequently, artificial honey composed of fructose, glucose, sucrose, and maltose, which mimics the sugar profile of honey produced by European honeybees, was used as a blanking solution in this study. To confirm that trehalulose had no impact on the FC assay, a 20% (*w*/*v*) trehalulose solution was also analysed following the methodology described in Section 2.6. It was found that trehalulose did not produce a detectable reaction in the modified assay. It can, therefore, be concluded that the TPC recorded in this study is an accurate reflection of the stingless bee honey’s total phenolic content.

Table 4 shows the average TPC values determined for the investigated samples. They ranged from 22.1 to 63.8 mg GAE/100 g, with an average of 37.8 ± 11.2 mg GAE/100 g. Sample H2 (produced by *Tetragonula hockingsi*) had the lowest value, while sample H4 (also a *Tetragonula hockingsi* honey) was found to have the highest phenolic content. Apart from being produced by different species of bees, the variation in TPC might also reflect the different botanical origins of the samples. Similar studies on TPC were performed by Zawawi et al. [17] on stingless bee honey, and values ranging from 88.3 to 132 mg GAE/100 g were reported for Australian honey and values from 22.3 to 54.2 mg GAE/100 g for Malaysian honey. Biluca et al. [33] reported TPC values between 11.0 and 38.9 mg GAE/100 g for Brazilian stingless bee honey. However, different analysis protocols, which might result in different TPC values, might also contribute to the differences recorded across these studies.

### 3.5. Antioxidant Activity

The present study determined the antioxidant activity of the honey samples using FRAP and DPPH assays. The FRAP data for the investigated samples is presented in Table 4 and reveals a reducing capacity ranging from 2.07 to 9.10 mmol Fe^2+^/kg, with an average of 4.82 ± 1.87 mmol Fe^2+^/kg. Sample H1 (from *Tetragonula hockingsi*) was found to have the highest activity, while sample H2 (also a *Tetragonula hockingsi* honey) exhibited the lowest. These results align with those reported by Biluca et al. [33] for stingless bee honey from Brazil, which had FRAP values that ranged from 1.79 to 10.5 mmol Fe^2+^/kg. On the other hand, Alvarez-Suarez et al. [11] reported lower reducing capacity (38.54 µmol Fe^2+^/100 g) for Cuban stingless bee honey.

The average DPPH radical scavenging activity of the investigated samples is also presented in Table 4. Across the analysed samples, a mean radical scavenging activity of 3.47 mmol TE/kg was found, ranging from 0.704 (sample H2) to 6.67 (sample H1) mmol TE/kg, both from *Tetragonula hockingsi* honey. Ávila et al. [46], Alvarez-Suarez et al. [11], and Mat Ramlan et al. [15] also reported DPPH radical scavenging activity for stingless bee honey from Brazil, Cuba, and Malaysia/Australia, respectively. Taken together, these findings indicate that stingless bee honey, including Australian stingless bee honey, exhibits reducing capacity and free radical-scavenging activity and, thus, antioxidant activity.

Several studies have associated the antioxidant activity found in honey with the presence of phenolic compounds and other substances [13,47,48]. Consistent with the literature, a strong correlation factor (0.846) between FRAP activity and total phenolic content was observed, as well as between DPPH and TPC values (0.932), indicating the significant contribution of phenolic compounds to the antioxidant activity of the investigated honey samples. Additionally, a high correlation (0.952) between DPPH and FRAP antioxidant activity was also noted.

The variations in the antioxidant activities among the honey samples may be attributed to the differences in the phenolic contents and different types of phenolic compounds present, as each phenolic compound exhibits different scavenging activity and reducing capacity [45]. Factors such as botanical origin, geographical area, time of harvest, and environmental conditions can influence the type and concentration of phenolic compounds and, thereby, the antioxidant activity of honey [46,48,49]. In addition, other factors such as handling practices, processing technologies, and storage conditions can further affect the antioxidant properties of the honey [50].

## 4. Conclusions

The study analysed various physicochemical attributes of Australian stingless bee honey, including pH, moisture, soluble solids, electrical conductivity, and sugar content. Results revealed that many of these parameters did not meet the current Codex Alimentarius standards for honey. The consumer evaluation detected a sour taste in Australian stingless bee honey. The assessment of the honey’s bioactivity, expressed in its total phenolic content and antioxidant activity, indicates its potential as a readily available source of natural antioxidants. The findings of this study highlight the uniqueness of Australian stingless bee honey, although significant variability in its physicochemical features can be noted, likely attributable to differences in geographical location and bee species involved in its production, as well as in its floral origin and time of harvest, which seem to affect its trehalulose content. The data generated from this research might prompt the development of specific international regulations for stingless bee honey. In addition, this research contributes to the dissemination of information on Australian stingless bee honey as a product with properties that might be beneficial to human health.

## Figures and Tables

**Table 1:** Honey samples.

Species	Sample	District Area	Harvest Date
*Tetragonula carbonaria*	C1 (*n* = 5)	Burpengary East, Property A	May 2022
	C2 (*n* = 5)	Burpengary East, Property B	May 2022
	C3 (*n* = 5)	Burpengary East, Property A	September 2022
	C4 (*n* = 3)	Burpengary East, Property B	September 2022
	C5 (*n* = 6)	Burpengary East, Property A	November 2022
	C6 (*n* = 2)	Burpengary East, Property B	November 2022
	C7 (*n* = 1)	Brighton	September 2021
	C8 (*n* = 1)	Tarragindi	March 2022
*Tetragonula hockingsi*	H1 (*n* = 2)	Burpengary East, Property A	May 2022
	H2 (*n* = 2)	Burpengary East, Property A	September 2022
	H3 (*n* = 2)	Burpengary East, Property A	November 2022
	H4 (*n* = 1)	Tarragindi	Not provided
	H5 (*n* = 1)	Brighton	September 2020

**Table 2:** Physicochemical characteristics of the stingless bee honey samples.

Sample	Moisture (%*w*/*w*)	Soluble Solids (°Brix)	Electrical Conductivity (mS/cm)	pH
C1	26.7 ± 1.71 ^a,b^	73.3 ± 1.71 ^a,b^	1.34 ± 0.108 ^a^	3.85 ± 0.0619 ^a,b^
C2	27.1 ± 0.868 ^a,b^	72.9 ± 0.868 ^a,b^	1.29 ± 0.275 ^a^	3.69 ± 0.0391 ^a,b^
C3	27.5 ± 0.856 ^a,b^	72.5 ± 0.856 ^a,b^	1.62 ± 0.170 ^a^	4.64 ± 0.244 ^a,b,c,d^
C4	28.3 ± 0.361 ^a^	71.7 ± 0.361 ^a^	1.41 ± 0.0961 ^a^	3.60 ± 0.0493 ^a^
C5	27.1 ± 0.501 ^a,b^	72.9 ± 0.501 ^a,b^	1.41 ± 0.165 ^a^	4.91 ± 0.728 ^b,c,d^
C6	27.3 ± 0.283 ^a,b^	72.7 ± 0.283 ^a,b^	1.24 ± 0.226 ^a^	3.89 ± 0.00 ^a,b^
C7	27.9 ± 0.00 ^a,b^	72.1 ± 0.00 ^a,b^	1.52 ± 0.00 ^a^	3.73 ± 0.00 ^a,b^
C8	27.3 ± 0.00 ^a,b^	72.7 ± 0.00 ^a,b^	2.15 ± 0.00 ^b^	3.72 ± 0.00 ^a,b^
H1	29.9 ± 0.919 ^a^	70.2 ± 0.919 ^a^	1.53 ± 0.0283 ^a^	3.92 ± 0.0636 ^a,b^
H2	29.4 ± 2.05 ^a^	70.7 ± 2.05 ^a^	1.63 ± 0.0212 ^a,b^	5.90 ± 0.912 ^d^
H3	27.2 ± 1.98 ^a,b^	72.8 ± 1.98 ^a,b^	1.53 ± 0.247 ^a^	5.51 ± 0.785 ^c,d^
H4	24.9 ± 0.00 ^b^	75.1 ± 0.00 ^b^	1.62 ± 0.00 ^a^	4.38 ± 0.00 ^a,b,c^
H5	28.8 ± 0.00 ^a^	71.2 ± 0.00 ^a^	1.46 ± 0.00 ^a^	3.69 ± 0.00 ^a,b^

Values are expressed as mean ± standard deviation. Different superscript letters in the same column denote significant differences (ANOVA, *p* < 0.05).

**Table 3:** Main sugar profile of stingless bee honey samples.

Sample	Trehalulose (g/100 g)	Fructose (g/100 g)	Glucose (g/100 g)	Sucrose (g/100 g)	F/G	F/T	G/T
C1	26.4 ± 4.46 ^c,d^	15.7 ± 2.72 ^b^	8.37 ± 2.34 ^b^	Not detected	1.87	0.593	0.317
C2	30.7 ± 5.04 ^d^	10.2 ± 2.64 ^a^	4.62 ± 1.43 ^a^	Not detected	2.20	0.331	0.150
C3	16.2 ± 2.25 ^a,b^	26.3 ± 1.81 ^e,f,g^	19.7 ± 1.66 ^d,e^	Not detected	1.33	1.63	1.22
C4	21.3 ± 2.83 ^b,c^	20.8 ± 2.12 ^c,d^	13.5 ± 1.07 ^c^	Not detected	1.54	0.977	0.636
C5	11.4 ± 2.03 ^a^	28.6 ± 1.84 ^f,g,h^	23.4 ± 1.65 ^f^	Not detected	1.22	2.51	2.06
C6	15.7 ± 0.493 ^a,b^	22.2 ± 0.331 ^c,d,e^	17.5 ± 1.16 ^d^	Not detected	1.27	1.42	1.12
C7	15.8 ± 0.735 ^a,b^	24.2 ± 0.246 ^d,e,f^	16.8 ± 0.350 ^c,d^	Not detected	1.44	1.54	1.07
C8	27.1 ± 0.786 ^c,d^	11.1 ± 0.370 ^a^	4.31 ± 0.175 ^a^	Not detected	2.56	0.408	0.159
H1	25.5 ± 4.84 ^c,d^	17.7 ± 4.35 ^b,c^	9.86 ± 2.95 ^b^	Not detected	1.79	0.694	0.387
H2	12.5 ± 2.67 ^a^	30.0 ± 1.04 ^g,h^	22.5 ± 0.526 ^e,f^	Not detected	1.34	2.39	1.79
H3	10.0 ± 4.24 ^a^	31.1 ± 2.56 ^h^	24.0 ± 3.08 ^f^	Not detected	1.30	3.09	2.39
H4	12.0 ± 0.257 ^a^	29.6 ± 0.882 ^g,h^	17.7 ± 0.379 ^d^	Not detected	1.67	2.48	1.48
H5	11.2 ± 0.399 ^a^	26.7 ± 0.926 ^e,f,g,h^	16.4 ± 0.437 ^c,d^	Not detected	1.62	2.38	1.46

Values are expressed as mean ± standard deviation. Different superscript letters in the same column denote significant differences (ANOVA, *p* < 0.05).

**Table 4:** Total phenolic content and antioxidant activity of the stingless bee honey samples.

Sample	TPC (mg GAE/100 g)	FRAP (mmol Fe^2+^/kg)	DPPH (mmol TE/kg)
C1	40.2 ± 5.17 ^d,e^	6.23 ± 0.656 ^g,h^	4.30 ± 1.06 ^e^
C2	36.9 ± 7.81 ^c,d,e^	4.52 ± 0.639 ^d,e,f^	3.00 ± 0.543 ^c,d,e^
C3	25.3 ± 2.36 ^a,b^	3.07 ± 0.284 ^a,b^	1.48 ± 0.353 ^a,b^
C4	29.9 ± 1.05 ^a,b,c^	3.86 ± 0.517 ^b,c,d^	2.77 ± 0.243 ^b,c,d^
C5	33.3 ± 4.70 ^b,c,d^	3.35 ± 0.649 ^b,c^	2.76 ± 0.754 ^b,c,d^
C6	34.7 ± 1.47 ^c,d,e^	4.33 ± 0.333 ^c,d,e^	3.70 ± 0.378 ^d,e^
C7	43.8 ± 0.363 ^e^	5.93 ± 0.211 ^g,h^	3.93 ± 0.125 ^d,e^
C8	37.2 ± 0.201 ^c,d,e^	5.25 ± 0.0609 ^e,f,g^	3.78 ± 0.195 ^d,e^
H1	53.7 ± 4.85 ^f^	9.10 ± 0.388 ^i^	6.67 ± 0.834 ^f^
H2	22.1 ± 2.94 ^a^	2.07 ± 0.776 ^a^	0.704 ± 0.525 ^a^
H3	31.2 ± 4.45 ^a,b,c,d^	3.02 ± 0.828 ^a,b^	2.03 ± 1.04 ^a,b,c^
H4	63.8 ± 1.44 ^g^	6.42 ± 0.259 ^h^	5.95 ± 0.203 ^f^
H5	39.4 ± 0.0644 ^d,e^	5.56 ± 0.0765 ^f,g,h^	4.04 ± 0.0865 ^d,e^

Values are expressed as mean ± standard deviation. Different superscript letters in the same column denote significant differences (ANOVA, *p* < 0.05).

## Data Availability

The original contributions presented in the study are included in the article, further inquiries can be directed to the corresponding author.
